# 1806. The gap in the amount of monthly antimicrobial use according to the data source: Electronic Health Record Data vs. National Health Insurance Claim Data in Korea

**DOI:** 10.1093/ofid/ofac492.1436

**Published:** 2022-12-15

**Authors:** Bongyoung Kim, Jung Mi Chae, Dong-Sook Kim, Hyunki Woo, Changhui Kim, Hong Bin Kim, Hyung-Sook Kim, Sun Hee Park, Su Jin Jeong, Young Uh, Song Vogue Ahn, Yoon Soo Park, Jun Yong Choi

**Affiliations:** Department of Internal Medicine, Hanyang University College of Medicine, Seongdong-gu, Seoul-t'ukpyolsi, Republic of Korea; Health Insurance Review & Assessment Service, Wonju, Kangwon-do, Republic of Korea; Health Insurance Review & Assessment Service, Wonju, Kangwon-do, Republic of Korea; Evidnet Inc., Seongnam, Kyonggi-do, Republic of Korea; Evidnet Inc., Seongnam, Kyonggi-do, Republic of Korea; Seoul National University Bundang Hospital, Seongnam, Kyonggi-do, Republic of Korea; Seoul National University Bundang Hospital, Seongnam, Kyonggi-do, Republic of Korea; College of Medicine, The Catholic University of Korea, Seoul, Seoul-t'ukpyolsi, Republic of Korea; Yonsei University College of Medicine, Seoul, Seoul-t'ukpyolsi, Republic of Korea; Yonsei University Wonju College of Medicine, Wonju, Kangwon-do, Republic of Korea; Ewha Womans University, Seoul, Seoul-t'ukpyolsi, Republic of Korea; Department of Internal Medicine, Division of Infectious disease, Yongin Severance Hospital, Yonsei University College of Medicine, Yongin, Kyonggi-do, Republic of Korea; Yonsei University College of Medicine, Seoul, Seoul-t'ukpyolsi, Republic of Korea

## Abstract

**Background:**

Korea has single health insurance system and insurance claim information on almost all medical practices in Korean hospitals is collected and processed by the Health Insurance Review and Assessment Service (HIRA). Since information about prescription of almost all hospitals is available in National Health Insurance (NHI) claim data, recently established the Korea National Antimicrobial Use Analysis System (KONAS) has been using NHI claim data as data source. The purpose of this study is to validate the accuracy of NHI claim data.

**Methods:**

Data on all antimicrobial agents prescribed in four tertiary-care hospitals in Korea between January 2019 and December 2019 were obtained using NHI claim data extracted by HIRA and data extracted by common data model based on electronic health record (EHR) in each hospital. Antibiotics and antifungal agents according to the Anatomical Therapeutic Chemical class J01 and J02 were included while antiviral, antitubercular, antiparasitic, and topical antimicrobial agents were excluded. Antimicrobial consumption was measured as days of therapy (DOT) and standardized to per 1,000 patient-days. The ratio of monthly antimicrobial consumption calculated using the NHI claim data compared to that calculated using the common data model was demonstrated (HIRA/EHR ratio).

**Results:**

The monthly HIRA/EHR ratio of broad-spectrum antibiotics predominantly used for hospital-onset infections was 1.08-1.12 and that of broad-spectrum antibiotics predominantly used for community-acquired infections was 1.11-1.21. The monthly HIRA/EHR ratio of other antimicrobial classes are as follows: antibacterial agents predominantly used for resistant gram-positive infections 1.15-1.31, narrow-spectrum beta-lactam agents 1.00-1.05, antifungal agents predominantly used for invasive candidiasis 1.00-1.27, and antibacterial agents predominantly used for extensive antibiotic-resistant gram-negative bacteria 0.70-1.09.

**Figure:**
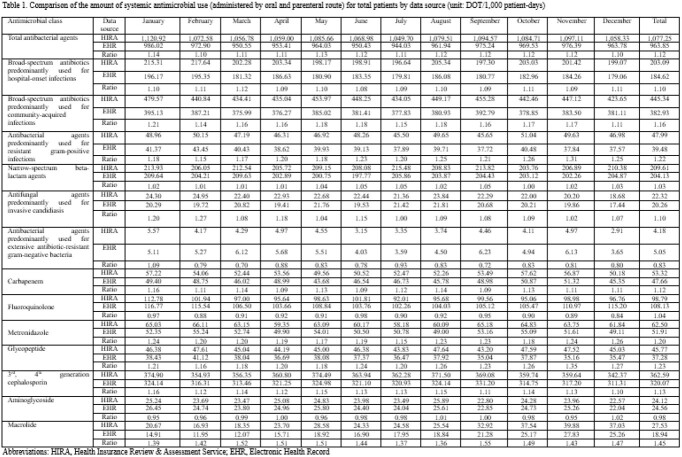

**Conclusion:**

The monthly antimicrobial consumption calculated using NHI claim data differs from that calculated using EHR data by up to 30%. It would be desirable to establish a system that can analyze and monitor antimicrobial consumption using EHR data in each hospital in Korea in the future.

**Disclosures:**

**Hyunki Woo, BS**, Evidnet Inc.: Employee **changhui Kim, BS**, Evidnet Inc.: Employee.

